# Introgression potential between safflower (*Carthamus tinctorius*) and wild relatives of the genus *Carthamus*

**DOI:** 10.1186/1471-2229-11-47

**Published:** 2011-03-14

**Authors:** Marion Mayerhofer, Reinhold Mayerhofer, Deborah Topinka, Jed Christianson, Allen G Good

**Affiliations:** 1 Department of Biological Sciences, University of Alberta, Edmonton, AB, Canada, T6G 2E9; 2 Department of Agricultural, Food, and Nutritional Science, University of Alberta, Edmonton, Alberta, T6G 2P5, Canada

## Abstract

**Background:**

Safflower, *Carthamus tinctorius*, is a thistle that is grown commercially for the production of oil and birdseed and recently, as a host for the production of transgenic pharmaceutical proteins. *C. tinctorius *can cross with a number of its wild relatives, creating the possibility of gene flow from safflower to weedy species. In this study we looked at the introgression potential between different members of the genus *Carthamus*, measured the fitness of the parents versus the F1 hybrids, followed the segregation of a specific transgene in the progeny and tried to identify traits important for adaptation to different environments.

**Results:**

Safflower hybridized and produced viable offspring with members of the section *Carthamus *and species with chromosome numbers of n = 10 and n = 22, but not with n = 32. The T-DNA construct of a transgenic *C. tinctorius *line was passed on to the F1 progeny in a Mendelian fashion, except in one specific cross, where it was deleted at a frequency of approximately 21%. Analyzing fitness and key morphological traits like colored seeds, shattering seed heads and the presence of a pappus, we found no evidence of hybrid vigour or increased weediness in the F1 hybrids of commercial safflower and its wild relatives.

**Conclusion:**

Our results suggest that hybridization between commercial safflower and its wild relatives, while feasible in most cases we studied, does not generate progeny with higher propensity for weediness.

## Background

The genus *Carthamus *is a diverse group of plants within the Asteraceae and is of interest due to the commercial growth of one member, *C. tinctorius *(safflower) as well as for its potential as a model system to examine the introgression of agronomic and weedy traits across species boundaries and to study the invasiveness of wild relatives of a crop. Safflower is grown in several countries as an oilseed crop and for birdseed and is being evaluated as a crop platform for molecular farming [1]. The different species of *Carthamus *have been classified into several different grouping systems by different taxonomists. Estilai and Knowles [2] originally placed 13 species in the genus *Carthamus *into five sections, based on chromosome numbers. Lopez-Gonzalez [3] rearranged the 15 species that he identified into three sections (*Carthamus*, *Odonthagnathis *and *Atractylis*), to match the understanding of the relationships between the species and their chromosome numbers. In the scheme proposed by Vilatersana *et al. *[4], the section *Carthamus *contains the species with 12 sets of chromosomes including *C. tinctorius*, *C. palaestinus *and *C. oxyacanthus*. The section *Atractylis *(n = 10, 11, 22, 32) contains all other species in the genus including the noxious weeds *C. lanatus *(n = 22) and *C. leucocaulos *(n = 10). There are still some species with uncertain placement within the groups, such as *C. nitidus *[5]. In this report, we have chosen to use the classification system of Lopez-Gonzalez. Elucidating species relationships within *Carthamus *has been challenging. There are low levels of genetic variation despite clear morphological differences between species [4,6]. Random amplified polymorphic DNA markers [RAPDs; 4] and conserved, intron-spanning PCR markers [7] have been utilized to address species relationships. Recently, because of low reproducibility of RAPD marker results [8] and conflict between published data sets, we have utilized microsatellite markers to analyze species relationships [6].

We have been using the genus *Carthamus *as a model system to study the introgression of traits across species boundaries and the extent to which these traits provide adaptive benefits. Several species of weedy relatives are growing in the same areas as the commercial crops and have the potential to cross and produce fertile offspring with safflower. These include *C. lanatus *(woolly distaff thistle, saffron thistle), *C. leucocaulos *(glaucous star thistle, white-stem/yellow distaff thistle), and *C. oxyacanthus *(jewelled distaff thistle, wild safflower). The genus is native to the Middle East; however, its distribution has expanded into many countries across the world including Australia and North America [9]. Both *C. lanatus *and *C. leucocaulos *are considered noxious weeds in California and Australia. In Australia, *C. lanatus *has become a weed after it was introduced from the Mediterranean. It has spread throughout the continent [10] and is currently considered the most economically damaging thistle species in New South Wales [11]. There are other thistle species within the family Asteraceae and some of them are noxious weeds, including spotted knapweed (*Centaurea maculosa*), diffuse knapweed (*Centaurea diffusa*) and star thistle (*Centaurea solstitialis*). These species are highly invasive, particularly in drier Prairie climates. In Canada, knapweed is now recognized as a major invasive weed, causing significant damage to a number of Prairie agroecosystems [12].

Hybridization of safflower with sympatric wild relatives has probably played a significant role in the evolution of *Carthamus *and cultivated safflower in the Mediterranean [13-15]. For example, the hexaploid noxious weeds *C. creticus *and *C. turkestanicus *are allopolyploids resulting from the hybridization of a tetraploid ancestor (*C. lanatus*) with a diploid progenitor lineage (*C. leucocaulos *and *C. glaucus*, respectively) [15]. The fact that these species intercross and that some of the relatives are weedy, leads to concerns about transgene escape from cultivated *C. tinctorius *plants and the potential for commercial safflower to cross with its weedy relatives and become feral or "de-domesticated".

The evolution of agricultural weeds from wild species is a recurring pattern in the history of agriculture, with plants from numerous families evolving weedy genotypes that thrive in cultivated areas [16]. This is not surprising, given the evidence that 12 of the 13 most important food crops hybridize with at least one wild relative within their range [Reviewed in 17]. Typically, during the development of crop plants a number of traits are commonly selected for, including high germination rates, yield, oil profile, earliness and developmental consistency. Similarly, when a wild species evolves into an agricultural weed, a number of important adaptations occur, including rapid seedling growth, high competitive ability and increases in both seed output and dispersal [18]. These adaptations are relevant for several reasons. First, these traits are encoded by multiple independent genes and the evolution of similar traits in different species is of interest from a comparative genetics viewpoint [19]. Second, the adaptations often result from the transfer of crop genes that provide specific life history traits for the hybrid to become a noxious weed. A particularly clear example of this has been the transfer of transgenes that encode herbicide resistance to create weeds with herbicide tolerance [20,21].

In addition to concerns about transgene escape there are now speculations that certain traits will allow invasive species to capitalize on different elements of global climate change [22].

In this paper, we describe which members of the *Carthamus *tribe can hybridize with cultivated safflower, determine whether the hybrid plants have a higher fitness than the *C. tinctorius *parent and look at the segregation of a herbicide resistance transgene in an interspecific cross. Finally, we analyze traits potentially important for adaptation to specific biotic environments.

## Results

### Crossing success and fitness of hybrids

Table 1 outlines the total number of seeds harvested and the success rate (# seeds produced/# crosses attempted × 100) of each cross. The success rate of controlled crosses between a transgenic *C. tinctorius *(Centennial) and other *Carthamus *species varied from 0% to 67%, compared to the *C. tinctorius*/*C. tinctorius *control cross of 40%. We are aware that some of the *Carthamus *lines obtained from the USDA might be fairly inbred and therefore may have given low seed set due to inbreeding depression.

**Table 1:** Success of crosses between *C. tinctorius *and relatives of the *Carthamus*-*Carduncellus *complex

	Male parent							
	
Female parent	*C. oxyacanthus^c^*	*C. palaestinus*	*C. leucocaulos*	*C. glaucus*	*C. lanatus*	*C. turkestanicus*	*C. creticus*	*C. tinctorius*
***C. oxyacanthus *(n = 12)^c^**	6% (6)							2/14% (32)
***C. palaestinus *(n = 12)**		15% (9)						38% (84)
***C. leucocaulos *(n = 10)**			68% (28)					67% (92)^a^
***C. glaucus *(n = 10)**				16% (6)				18% (40)
***C. lanatus *(n = 22)**					49% (28)			17% (26)^a^
***C. turkestanicus *(n = 32)**						54% (58)		0.3% (1)^b^
***C. creticus *(n = 32)**							55% (22)	2% (3)
***C. tinctorius *(n = 12)**	15/23% (82)	31% (86)	14% (23)^a^	41% (67)	29% (55)^a^	0%	0%	40% (131)

Calculation of success rate: (# seeds produced/# crosses attempted) × 100. Number in brackets is the number of seeds harvested.

^a ^sterile F1 plants

^b ^no F1 plants

^c ^*C. oxyacanthus *genotypes PI 426427/PI 426477

Crosses with species in the section *Carthamus *(n = 12; *C. oxyacanthus *and *C. palaestinus*) generally worked, regardless of whether *C. tinctorius *was the male or female parent. Crosses worked equally well with *C. palaestinus *as either the female (38%) or the male (31%) parent. Two different accessions of *C. oxyacanthus *(PI 426427, PI 426477) had a low success rate as the female parents (2% and 14%) with somewhat higher values as the male parents (15% and 23%). A nontransgenic variety of *C. tinctorius *(Centennial) was also crossed with two other accessions of *C. oxyacanthus*. These crosses worked well with *C. oxyacanthus *as female parent (56% and 30%) and a bit less efficient as the male parent (21% and 42%; data not shown).

Crosses between species of the section *Odonthagnathis *(n = 10; *C. leucocaulos *and *C. glaucus*) and *C. tinctorius *were relatively successful, ranging from 14% to 67% success rate. The cross with *C. glaucus *produced fertile F1 plants; however, the cross with *C. leucocaulos *resulted in sterile offspring. Our recent data and similar findings by other laboratories have raised doubts about the identity of the *C. glaucus *samples that are being distributed by USDA Pullman, WA, i.e. these seeds might in fact not be from *C. glaucus *but from a species with n = 12.

For the section *Atractylis *(n = 22, 32; *C. lanatus, C. turkestanicus, C. creticus*), the cross between *C. lanatus *(n = 22) and *C. tinctorius *worked well with *C. lanatus *as male parent (29%), with a lower success rate as female parent (17%). However, all F1 plants from this cross were sterile.

For *C. turkestanicus*, two different genotypes were used (PI 426180, PI 426426). Only one seed was harvested, giving a success rate of 0.3%. We did not determine whether this seed was truly a hybrid, would germinate and produced viable F1 plants.

*C. creticus *as female parent gave a 2% success rate, and 0% as male parent, therefore it was assumed that crosses between these species were unlikely to work. In summary, crosses with members of the section *Atractylis *were successful for *C. lanatus *(n = 22) but failed for *C. creticus *and *C. turkestanicus *(both n = 32).

A number of seeds from all crosses, except *C. creticus *and *C. turkestanicus*, were imbibed to determine the germination rates and to produce F1 plants for further analysis (Table 2 and 3). In total 197 F1 plants were generated and all, except two self-pollinated individuals, were true hybrids as verified by species-specific microsatellite markers and antibody based test strips. The hybrid plants were subsequently selfed for the generation and analysis of F2 seeds (Table 3).

**Table 2:** Fitness and transgene deletion

Species	Parental fitness	F1 fitness	F1 germination rate (%)	Deletion of transgene in F1s
*C. glaucus *(n = 10)	0.46	1.22	75	21% (15/72)
*C. leucocaulos *(n = 10)	13.41	0	80	0% (0/62)
*C. oxyacanthus *(n = 12)^a^	1.27/0.05	1.06/1.21	32/36	0% (0/34)
*C. palaestinus *(n = 12)	2.21	1.60	90	0% (0/9)
*C. tinctorius *(n = 12)	1.00	-	-	0% (0/67)
*C. lanatus *(n = 22)	6.51	0	33	0% (0/18)

Calculation of fitness: Parental fitness = (# parental seed/# Centennial seed), F1 Fitness = (# F2 seed/# Centennial seed).

^a ^*C. oxyacanthus *genotypes PI 426427/PI 426477

**Table 3:** Domestication and ferality characteristics of F1 hybrids between *C. tinctorius *and wild relatives

		F1 hybrids with *C. tinctorius*					
		
Plant stage	Trait	*C. oxy.(27)*^a^	*C. oxy.(77)*^b^	*C. pal.*	*C. leuc.*	*C. glauc.*	*C. lan.*
**Seed**	Pappus (T♀/T♂)	No/No	No/No	No/Some	No/Yes	No/No	No/Yes
	Seed color (T♀/T♂)	W-T/St-W	W-T/St-W	C-B/W-C	W-B/B	C-T/W-C	T-B/T-B
	mg/seed (T♀)	38.7 ± 10.9	38.7 ± 7.9	53.3 ± 4.8	31.0 ± 6.7	47.3 ± 5.3	21.1 ± 4.7
	mg/seed (T♂)	13.0 ± 0.0	11.7 ± 2.0	51.1 ± 17.1	11.6 ± 1.1	36.3 ± 3.8	17.8 ± 1.0
	Seed weight (% Centennial, T♀/T♂)	71.4%/24.0%	71.4%/21.6%	98.3%/94.3%	57.2%21.4%	87.3%/67.0%	38.9%/32.8%
	Germination rate	32%	36%	90%	80%	75%	33%
							
**Rosette**	Number of spines	29.0 ± 4.6	31.5 ± 8.4	25.7 ± 7.7	102.6 ± 17.3	21.8 ± 3.4	104.7 ± 10.8
							
**Bolting**	Rosette period (days)	22.7 ± 2.0	23.1 ± 0.6	19.4 ± 3.7	28.9 ± 2.0	20.0 ± 0.9	36.3 ± 1.2
							
**Inflorescence**	Days to flowering	57.4 ± 2.4	61.5 ± 1.9	60.8 ± 3.9	62.9 ± 3.5	61.1 ± 1.5	71.7 ± 0.6
	Days of flowering	66.6 ± 7.5	72.1 ± 17.7	< 48.1	96.6 ± 9.6	45.2 ± 10.2	76.7 ± 11.1
	Number of branches	10.1 ± 1.2	12.5 ± 1.9	7.0 ± 1.5	10.3 ± 1.7	10.0 ± 2.1	6.7 ± 1.2
							
**Mature head**	Shattering	low to high	low to high	No	n/a	Some	n/a
	Flower heads/plant	41.7 ± 5.1	41.8 ± 8.2	9.7 ± 1.4	79.0 ± 8.7	11.3 ± 4.2	42.3 ± 12.3
							
**F2 seed**	F2 seeds/plant (#)	179 ± 88	205 ± 141	271 ± 64	0	205 ± 47	0
	F2 seed/plant (g)	6.2 ± 2.8	6.8 ± 4.5	12.5 ± 2.5	0	10.5 ± 2.1	0
	F2 mg/seed	35.6 ± 3.7	34.5 ± 4.5	47.4 ± 9.0	n/a	51.6 ± 5.6	n/a

Phenotypes were obtained from three to ten F1 hybrids from each cross.

^a ^*C. oxyacanthus *genotype PI 426427, ^b ^*C. oxyacanthus *genotype PI 426477, T♀: *C. tinctorius *was the female parent, T♂: *C. tinctorius *was the male parent.

Seed color: (W) white, (C) cream, (T) tan, (B) brown, (St) brown/brown striped. Rosette spine number: Maximum number of spines per leaf. Shattering: (low to high) variable frequency of shattering.

While there are a number of ways of calculating parental and F1 fitness, our calculation of fitness was based solely on the total seed set per plant, given as a fraction of the seed set of the commercial cultivar Centennial (Table 2). However, we note that some of the Carthamus lines are likely to be fairly inbred, due to repeated selfing and seed collection, which occurs as a result of the way the USDA maintains its lines. Thus, some selfs may have given low seed set due to inbreeding depression, whereas outcrossing relieves this, resulting in higher seed set.

Parental fitness varied from 0.05 (for one *C. oxyacanthus *genotype) to 13.41 (for *C. leucocaulos)*, compared to the Centennial parental fitness (1.00). The F1 fitness was zero for *C. lanatus *and *C. leucocaulos *as all of the self-pollinated F1 plants (11 and 18 plants, respectively) had a very low amount of pollen and none of them produced any seed.

### Domestication and weedy characteristics of *C. tinctorius *and the wild relatives

After analyzing the key descriptors for safflower [23], we developed a list of traits that could potentially be associated with domestication or weediness and analyzed them in the parental species (Table 4) and the F1 hybrids (Table 3). These included seed weight, seed color, presence of a pappus, number of seeds produced, time at rosette stage, spininess, time to flowering, time of flowering and shattering versus non-shattering heads. Three key morphological traits that may be associated with weediness are colored seeds, shattering seed heads and the presence of a pappus. *Carthamus lanatus*, *C. leucocaulos*, *C. turkestanicus *and *C. creticus *all have a pappus on their seeds and are shattering, and most of the wild species have seeds that are tan, brown or brown striped, all of which should help in seed dispersal and in reducing seed predation. Additionally, most of the wild species studied had much higher numbers of seeds per plant.

**Table 4:** Domestication and ferality characteristics of parental species within the *Carthamus *family

		Parental species							
		
Plant stage	Trait	*C. tinct.*	*^a^C. oxy.*	*C. pal.*	*C. leuc.*	*C. glauc.*	*C. lan.*	*^b^C. turk.*	*C. cret.*
**Seed**	Pappus	No	No	Some	Yes	Some	Yes	Yes	Yes
	Seed color	W	St	W	B	W-T	T-B	B	B
	mg/seed	51.4 ± 4.5	9.9/13.1	38.6 ± 5.6	9.1 ± 0.3	44.4 ± 4.4	32.5 ± 3.7	44.5/49.8	25.8 ± 2.1
	Germination rate	95%	50%/75%	92%	100%	44%	100%	39%/100%	72%
	Seeds per plant	169 ± 55	215/8	373 ± 163	2267 ± 306	77 ± 43	1100 ± 100	860/543	753 ± 46
**Cotyledon**	Cotyledon size	55.2/21.7	51.5/8.5 55.6/11.5	51.6/21.6	34.5/14.1	51.0/20.9	70.8/29.7	55.1/27.9 59.2/28.8	61.4/21.9
**Rosette**	Leaf blade shape	oblanceolate	oblanceolate	oblanceolate	bipinnatifid	oblanceolate	pinnatifid	bipinnatifid	bipinnatifid
	Number of leaves	3.9 ± 0.6	12.5/15.7	11.0 ± 6.9	51.8 ± 1.8	8.8 ± 1.6	47.0 ± 5.8	74.0/53.8	34.6 ± 5.7
	Number of spines	12.9 ± 0.9	51.0/51.3	47.0 ± 6.9	300.0 ± 0.0	42.0 ± 7.13	460.0 ± 0.0	300.0/300.0	316.0 ± 43.4
	Spine location	1	4	3	4	2	4	4	4
**Bolting**	Rosette period (days)	12.4 ± 1.5	30.0/44.3	28.2 ± 6.2	85.0 ± 1.4	22.0 ± 1.1	74.6 ± 2.7	110.0/106.2	66.6 ± 8.1
	Number of branches	5.6 ± 0.9	11.0/19.3	8.0 ± 2.6	11.3 ± 1.5	17.3 ± 1.5	13.0 ± 3.6	11.0/22.3	27.7 ± 3.5
	Branch angle	I	I	I	A/I	I	S	S	S
	Branching position	upper 3/5	base to apex	upper 3/5	base to apex	base to apex	upper 4/5	upper 3/5	upper 3/5
**Inflorescence**	Days to flowering	69.1 ± 4.0	63.0/85.0	75.0 ± 13.0	153.2 ± 34.6	63.2 ± 1.9	122.8 ± 8.5	145.5/139.0	98.6 ± 4.6
	Days of flowering	36.3 ± 10.8	na/64.7	64.0 ± 19.2	99.3 ± 4.5	97.5 ± 3.5	54.7 ± 12.7	108.3/70.0	52.7 ± 7.0
	Heads per branch	5.0 ± 1.2	38.0/16.7	6.3 ± 4.9	68.3 ± 19.7	6.7 ± 2.9	26.0 ± 1.7	11.0/9.3	14.0 ± 1.7
**New flowers**	Corolla color (petals)	yellow	yellow	yellow	white-purple	yellow	yellow	light yellow	cream
**Mature head**	Shattering	No	Some/Some	No	Yes	No	Yes	Yes	Yes
	Flower heads per plant	9.0 ± 1.9	215.0/144.0	35.0 ± 19.9	413.3 ± 24.8	57.3 ± 21.7	126.0 ± 42.3	50.7/70.3	85.7 ± 9.2

Phenotypes were obtained from a minimum of five parental individuals.

^a ^*C. oxyacanthus *genotypes PI 426427/PI 426477, ^b ^*C. turkestanicus *genotypes PI 426180/PI426426.

C. tinct.: C. tinctorius, C. oxy.: C. oxyacanthus, C. pal.: C. palaestinus, C.leuc.: C. leucocaulos, C. glauc.: C. glaucus, C. lan.: C. lanatus, C. turk.: C. turkestanicus, C. cret.: C. creticus. Seed color: (W) white, (C) cream, (T) tan, (B) brown, (St) brown/brown striped. Cotyledon size: Length/Width in mm. Rosette spine number: Maximum number of spines per leaf. Rosette spine location: (1) distal 1/3 to 1/2, (2) distal 1/2 to 2/3, (3) distal 1/2 to all along margins, (4) tip and all along margins. Branch angle: (A) appressed (15° to 20°), (I) intermediate (20° to 60°), (S) spreading (60° to 90°). Heads per branch: Maximum number.

Other traits that may be related to weediness or invasiveness are a longer time at the rosette stage and to the start of flowering, as well as spininess. Shoot elongation is delayed for the weedy relatives and their time at the rosette stage was 1.8 to 6.9 times that of Centennial. For the F1 plants this was 1.6 to 2.9 times and it always fell between the two parents.

Time to flowering differed substantially between the species analyzed, with *C. leucocaulos *and *C. lanatus *showing the longest time. The values for the F1s fell between the two means of their parents.

There was a wide range of the number of selfed seeds per plant for the parental species, ranging from a few to over two thousand, although some individual plants did not produce any seeds at all. The wild relatives, particularly *C. lanatus *and *C. leucocaulos*, had many more seeds than Centennial. Having many smaller-sized seeds is probably a strategy used by these weeds to increase the dispersal and the probability that a viable seed will find a suitable environment. The two genotypes of *C. oxyacanthus *(PI 426427 and PI 426477) produced quite different amounts of seed (215 vs. 8) which may reflect some inherent self-incompatibility systems [24]. The seed set of the F1 plants also varied between the different crosses. However, the biggest variation was again seen between the progeny of the *C. oxyacanthus -C. tinctorius *cross.

The F1 germination rates of the two *C. oxyacanthus *accessions and of *C. lanatus *were about a third of *C. tinctorius*, which was almost 100%. They ranged from 32% to 36% and were also considerably lower than the germination rates of their weedy parents (50% to 100%). For *C. lanatus*, F1 seeds from the cross with *C. tinctorius *as the male parent had a considerably higher germination rate than from the reciprocal cross (not shown). *C. leucocaulos *and *C. palaestinus *parental and F1 seeds germinated at very similar rates (80% to 100%), whereas the *C. glaucus *F1s did significantly better than their weedy parent (75% vs. 44%). It should be noted however that fitness measurements on material which has very different histories (inbred for several generations, compared to a commercial cultivar or an F1 hybrid), are extremely difficult to compare, due to possible genetic effects associated with cultivar development, inbreeding or differences in hybrid breakdown in the F2 generation.

The seed weight of the F1 seeds either fell between that of Centennial (the parent with the larger seeds) and the wild relative, or it was lower. F1 seeds were always similar in color, size, shape and seed weight to the female parent of the cross, suggesting some degree of maternal inheritance to these traits (Table 3). The weight of the F2 seeds was between that of the two parents and there was no difference whether Centennial was the female or the male parent.

The weedy species are primarily shattering, which is likely to increase the dispersal rate of the seed. Since *C. lanatus, C. leucocaulos, C. turkestanicus and C. creticus *hybrids did not develop any F2 seed set, we have no data about this trait from these species. In the case of C. oxyacanthus, all the selfed F1 plants were shattering at variable degrees, suggesting a dominant trait; however, our data do not allow a more detailed genetic analysis.

Seed color was difficult to evaluate genetically but the weedy species and their F1 hybrids had mostly striped to brown seeds, that are clearly less visible against a soil, crop or grassland background.

The frequent presence of a pappus in *C. lanatus*, *C. leucocaulos*, *C. turkistanicus *and *C. creticus *indicates the value of this trait in these weedy species. We observed a pappus in the F1 seed when the weedy relative was the female parent, but not the reciprocal cross. Again, we could not analyze any F2 seeds for this trait in these crosses. Regardless of the trait measured, the F1 plants usually had a phenotype that was midway between the parents.

### Deletion of the transgene in specific F1 hybrids

**Figure 1:**
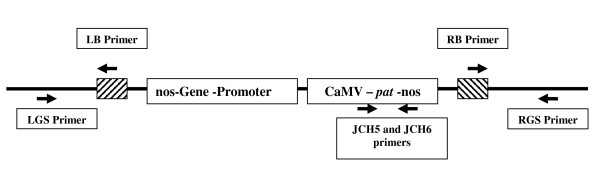
**The structure of the T- DNA construct and location of specific primers**. (LB and RB) T-DNA left and right border, (JCH5 and JCH6) *pat *gene flanking primers, (LGS and RGS) left and right genomic plant sequence, (*pat*) phosphinotricine acetyltransferase gene, (CAMV) Cauliflower Mosaic Virus promoter, (nos) Nopalin Synthase polyA site

The presence of the transgene in the F1 crosses was verified using an antibody strip test as well as T-DNA specific PCR primers, detecting the pat protein and *pat *gene, respectively. Additionally, the integrity of the left and right T-DNA border/plant DNA junctions were analyzed by PCR using the LB/LGS and RB/RGS primer pairs (Figure 1). We found that, with one exception, all crosses produced F1 offspring carrying the intact transgene. However, when *C. tinctorius *was crossed with *C. glaucus*, the pat protein and *pat *gene were absent in 21% of the progeny (Table 2). Instead, in those F1 plants the LGS and RGS primers amplified a single band of the same size as in the genomic region of the nontransgenic Centennial control, suggesting a complete deletion of the T-DNA construct. Table 5 shows the PCR and strip test results for four (out of 72 analyzed) F1 individuals, along with two controls plants. Hybrids glauc1 and glauc2 showed a deletion of the T-DNA construct, whereas glauc3 and glauc7 retained it. These patterns were consistent for all of the F1s we analyzed, i.e. the F1s that lacked the pat protein, the *pat *gene and the left and right T-DNA borders produced a wildtype Centennial band and *vice versa*. Since we separated the PCR products only by agarose gel electrophoresis, we were unable to determine whether there were any smaller deletions (<20bp) associated with the excision of the transgene. We did not observe any discernible morphological differences between these F1 plants and the ones carrying the transgene.

**Table 5:** PCR and antibody analysis of *C. tinctorius. *× *C. glaucus *F1 plants

Control/Cross	Sample Plant	RB/RGS	LB/LGS	JCH5/JCH6	LGS/RGS	pat Strip Test
						
**Non-transgenic Centennial**	Cent 10-2-4-1	-	-	-	**+**	-
**Transgenic Centennial**	T43	**+**	**+**	**+**	-	**+**
**T43 × glauc 51-5-1**	glauc 1	-	-	-	**+**	-
**T43 × glauc 51-5-1**	glauc 2	-	-	-	**+**	-
**T43 × glauc 51-5-3**	glauc 3	**+**	**+**	**+**	-	**+**
**T45 × glauc 51-5-1**	glauc 7	**+**	**+**	**+**	-	**+**

Cent 10-2-4-1: Non-transgenic *C. tinctorius *Centennial; T43 and T45: Transgenic *C. tinctorius *Centennial; glauc 51-5-1 and glauc 51-5-3: *C. glaucus *parent; glauc 1; glauc 1, glauc 2, glauc 3, glauc 7: C. glaucus F1 plants.

## Discussion

### Hybrid production

Safflower is considered one of humanities' oldest crops and has therefore been selected for domestication traits over several centuries [25]. It does have numerous wild relatives and gene transfer through interspecific hybridization may introduce weedy traits into the commercial crop, creating the potential for invasive hybrid populations [26-28]. Alternately, it can also provide an avenue for the transfer of novel traits from specially developed crops to wild populations. In the Old World there are a number of wild relatives that coexist with *C. tinctorius*, for example *C. palaestinus, C. persicus *and *C. oxyacanthus *[9,25,29,30]. In the New World, potential recipients of genes from cultivated safflower include four naturalized wild relatives, *C*. *creticus*, *C*. *lanatus*, *C*. *leucocaulos *and *C*. *oxyacanthus*. Of these, *C*. *oxyacanthus *and *C*. *creticus *have previously been shown to produce viable hybrid offspring with *C. tinctorius *[5]. We have now demonstrated that most of the wild relatives, which have 10 or 12 chromosomes, produce viable and fertile hybrids with *C. tinctorius*.

A number of hybrids from the different interspecific crosses are currently being advanced by selfing, as well as backcrossing to the wild parents. Monitoring of the fitness of subsequent generations will give us a better idea about the adaptive value of the incorporated crop genes. Also, other effects like hybrid breakdown [31] can be better recognized at later generations.

### Domestic versus weedy traits

Many of the traits detected in cultivated safflower such as Centennial have clearly been selected for by breeders. These include consistent white seeds, high germination rates, high yield and yield correlates (seed number and seed size), absence of a pappus, non-shattering, erect stature, etc. Therefore, the traits that may provide a selective advantage in an agricultural setting may not be selected for in nature or in an invasive weed. For example, while a high germination rate is valuable from a producer's perspective, delaying germination until a second year might allow a weedy genotype to germinate in a different environment, either in terms of the competitive environment (a different crop) or a different abiotic environment.

Several weedy relatives of *C. tinctorius *have been studied and hybrids between these relatives and safflower have been used to study the inheritance of a number of domestication traits [32,33]. The wild and weedy species *C. oxyacanthus, C. persicus *and *C. palaestinus *were shown to have seeds that are released by shattering, although in our study *C. palaestinus *was non-shattering. These species are homozygous dominant for the gene *Sh*, while cultivated safflower genotypes are homozygous recessive for this locus (*sh*) [29,30]. Another trait that alters seed dispersal in the Asteraceae is the presence of a pappus, a seed appendage for dispersal via water, wind and adherence to animal fur. Most of the seeds of safflower lack a pappus and when it is present, it is less than the length of the achenes. The gene controlling the presence of a pappus in *C. persicus *has been shown to be dominant (*P_*), while commercial safflower is homozygous recessive for this locus (*pp*) [30]. A third trait that has been genetically characterized is the duration of cultivated safflower's rosette stage, which is shortened by a single dominant gene (*ro*), reducing the maturity time of the crop, which might also affect the invasiveness of a particular genotype [30]. The longer rosette stage of both *C. persicus *and *C. oxyacanthus *helps their seeds to be dispersed in the field after harvest of the cereal crops they often grow among. Domestication traits such as large seed, reduced shattering, lack of pappus and short duration of the rosette stage ensure that the majority of safflower seeds are harvested. Reduced seed dormancy causes the seeds to germinate when planted so they are less likely to persist in the seed bank.

Data obtained from crosses of *C. tinctorius *with other species can be used as an initial indicator to predict the potential for hybridization and subsequent introgression of a gene from a cultivated crop into a weedy population and vice versa. Hybrids between safflower and wild relatives could potentially serve as a source of feral safflower populations but hybridization and introgression would require that both plants be sympatric in their distribution and flower at the same time. Our analysis of some of the traits that make *C. tinctorius *a commercial crop suggests that they are unlikely to provide any selective advantage.

### Segregation of a transgene in the hybrids

The movement of a specific transgene to the offspring was analyzed using a homozygous line with a single T-DNA insert. We observed that in all of the crosses, except one, the transgene acted as a normal Mendelian trait. However, in the *C. tinctorius *× *C. glaucus *cross, the transgene was deleted at a frequency of 21%.

The ultimate fate of a transgene in nature is affected by several factors including its frequency in the population, the probability that the gene will be transferred to the hybrid plant and finally, the selective advantage the gene confers to the new host species [34]. It seems unlikely that transgenes used for the production of Plant Made Pharmaceuticals (PMPs) would improve the viability or survival of feral safflower. In fact the only data we are aware of (McPherson M., unpublished data), suggest that the PMP trait used in these experiments reduces the fitness of the seed. Haygood *et al. *[35] have shown in their analysis that the likelihood of establishment and rate of spread of a transgene is governed primarily by the strength of selection, as opposed to the migration rate [35,36].

Several pieces of data now point to the unlikelihood of transgene escape, except when the transgene provides a selective advantage to the hybrid, e.g. herbicide tolerance. First, the outcrossing frequency of safflower is relatively low. Second, our data provide evidence of the selective deletion of transgenes in specific crosses, a phenomenon that we believe is the first of this kind in an interspecific cross. Third, the traits that breeders have selected for in cultivated safflower, like seed color, high germination rates, seed weight and non-shattering seed heads, appear unlikely to provide much of a selective advantage in competitive situations in nature, as they decrease both the seed number and dispersal characteristics of the hybrids. However, the adaptive value of crop genes can be different in backcross progeny growing under different environments. Given that the genus *Carthamus *includes several weeds such as *C. lanatus, C. leucocaulos *and *C. oxyacanthus*, it seems sensible to avoid growing transgenic safflower in geographical areas where feral species have been reported, e.g. drier regions including California and Australia, and areas where safflower is currently being grown as an oilseed crop.

## Conclusion

In this study, we report that commercial safflower will cross readily with different members of the same section (*Carthamus*) and several species with different chromosome numbers. All of these crosses produce F1 plants and most of them, particularly coming from wild relatives with n = 10 and n = 12, are viable and fertile. However, there is no evidence of hybrid vigour or other benefits provided to them.

Our analysis of some of the domestication traits that make *C. tinctorius *a commercial crop suggests that they are unlikely to provide any selective advantage when they are introgressed into wild relatives. Likewise, the transfer of a T-DNA construct from commercial safflower did not appear to have any visible effect on the hybrids.

The transgene was deleted in 21% of the hybrids from a specific cross, suggesting a negative selection mechanism against foreign DNA in some species.

## Methods

### Plant material

Additional File 1 provides a list of the germplasm used and the identifier number to allow the identification of the germplasm in our recent phylogenetic analysis as described in Bowles *et al. *[6]. The *C. tinctorius *parent in all crosses was the commercial safflower variety Centennial, which was homozygous for a transgene construct containing the *Phosphinothricin Acetyltransferase *(*pat*) gene as a selectable marker (Figure 1). Seeds for this line, as well as for a non-transgenic line of Centennial, were obtained from SemBioSys Genetics Inc. (Calgary, AB, Canada). The seed lots were tested for purity and homozygosity of the transgene as described by Christianson *et al. *[37]. For most accessions, seeds were germinated in soil. In those cases where no germination occurred in the first attempt, 0.3% gibberellic acid (GA_3_) in ddH_2_0 was added. Where possible, single seed descent was performed to reduce the level of genetic variability in the specific genotypes used in crossings. For interspecific crosses, plants were emasculated and hand pollinated, the flowers were bagged and the plants allowed to fully mature. For most crosses, three plants of each genotype were used as parents and reciprocal crosses were performed. In total, between 38 and 280 crosses were carried out for each species pair. In addition, positive control crosses (a cross with a plant of the same genotype) and negative control crosses (emasculation, but no pollination) were carried out. Once dried, seeds were harvested and stored for four to six months to allow for a break of dormancy. F1 seeds were then germinated in ddH_2_0 and sand and, where required, GA_3 _was added. Parents and F1 plants were evaluated for different growth parameters and for seed set. Plants were covered with micro perforated selfing bags and allowed to self-pollinate. A number of crosses are currently being evaluated at the F2 and BC1 stage.

### Genotypic analysis of plants

We used three procedures to genotype the F1 plants. Leaf tissue samples of the progeny were analyzed for the presence of the pat protein using antibody based test strips (Strategic Diagnostics Inc. 111 Pencader Drive, Newark DE). The presence and integrity of the *pat *gene and the T-DNA cassette was confirmed by PCR, using a combination of T-DNA and *pat *gene specific primers (Figure 1), as described by Christianson et al. [37]. Species-specific microsatellite markers [6, Mayerhofer R (unpublished results)] were used to ensure that the F1 plants were true hybrids.

Genomic DNA extractions from fresh or lyophilized leaf tissue were performed as described by Mayerhofer *et al. *[38]. The microsatellite loci were amplified using a modified protocol adapted from Schuelke [39]. PCR reactions contained 0.75 mM MgCl_2_, 0.2 mM dNTPs, 0.267 mM reverse and M13 labeled primers, 0.067 mM forward primer, 2.5 units of Taq DNA polymerase and 50-100 ng of template in 15 μl total volume. Thermocycling conditions were as follows: 94°C (5 min.); 30 cycles of 94°C (30sec), 56°C (45sec), 72°C (45sec); 9 cycles of 94°C (30 sec), 53°C (45 sec), 72°C (45 sec); ending with 72° for 10 minutes. Products from the PCR reactions were resolved on an ABI 3730 DNA Analyzer. Products were sized using Genemapper with the GeneScan 600 LIZ size standards (Applied Bioscience).

For those F1 plants where the pat protein was absent, the presence of specific components of the T-DNA cassette was determined. Figure 1 illustrates the key components of the T-DNA construct and the specific primers that were used to evaluate the F1 progeny.

Amplification of the T-DNA right border/plant DNA junction:

RB primer 5'-TATCCGCTCACAATTCCACAC-3'

RGS primer 5'-GGCAAGCCAAGCTATATCGTGACAAG-3'.

Amplification of the T-DNA left border/plant DNA junction:

LB primer 5'-TAAATTTGTAGGGATATCGTG-3'.

LGS primer 5'-CAAGTGGCTTTCTTTGTAAG-3'

Amplification of the *pat *gene:

JCH5, 5'-GATCTGGGTAACTGGTCTAACTGG-3'

JCH6, 5'-GTTGCAAGATAGATACCCTTGGTT-3'.

Each PCR reaction was carried out in 25 μl with 5 μl Q-solution (Qiagen), 2.5 μl 10 × PCR buffer, 3 mM MgCl_2_, 0.5 mM dNTPs, 0.5 mM of each primer, 40 ng of template and 2.5 units of Qiagen Taq polymerase. The cycle parameters were 95°C (10 min), followed by 35 cycles of 95°C (20 sec), 59°C (30 sec) and 72°C (45 sec), with a final elongation step of 5 minutes at 72°C.

## Authors' contributions

AGG conceived the investigation and wrote the paper with assistance from MM and RM. MM and DT performed the crosses and analyzed the plant material. RM carried out the microsatellite assays of the F1 hybrids. All authors have read and approved the final manuscript.

## Supplementary Material

Additional File 1 **List of germplasm used in study**. Accessions in bold were used in crosses Click here for file
