# Supplementary material for: Introgression potential between safflower (Carthamus tinctorius) and wild relatives of the genus Carthamus
Source: BMC Plant Biol. 2011 Mar 14;11:47. doi: 10.1186/1471-2229-11-47 (PMC3068944; doi:10.1186/1471-2229-11-47)
Supplement: Additional File 1 — List of germplasm used in study. Accessions in bold were used in crosses [file 1471-2229-11-47-S1.DOC]

**Additional File 1. List of germplasm used in study.** Accessions in bold were used in crosses

| **Species** | **Accession Number** | **Name** | **Originally from** | **Designation (Bowles et al.)** | **Germplasm database** |
| --- | --- | --- | --- | --- | --- |
| ***C. oxyacanthus*** | PI 235667 | Cal.54-40 | India |  | USDA Pullman, WA |
|  | PI 349687 |  | Pakistan |  | USDA Pullman, WA |
|  | PI 407602 | KN 71 | Turkey |  | USDA Pullman, WA |
|  | PI 426184 | K-1073 | Afghanistan |  | USDA Pullman, WA |
|  | **PI 426185** | K-1076 | Afghanistan | 185 | USDA Pullman, WA |
|  | **PI 426427** | K-1 | Pakistan | 427 | USDA Pullman, WA |
|  | **PI 426428** | K-2 | Pakistan |  | USDA Pullman, WA |
|  | **PI 426477** | K-641 | Pakistan | 477 | USDA Pullman, WA |
|  |  |  |  |  |  |
| ***C. turkestanicus*** | **PI 426180** | K-1029 | Afghanistan | 029 | USDA Pullman, WA |
|  | PI 426181 | K-1050 | Afghanistan |  | USDA Pullman, WA |
|  | PI 426425 | K-892 | Afghanistan |  | USDA Pullman, WA |
|  | **PI 426426** | K-908 | Afghanistan | 426 | USDA Pullman, WA |
|  |  |  |  |  |  |
| ***C. lanatus*** | PI 202728 |  | Belgium |  | USDA Pullman, WA |
|  | PI 235666 |  | Portugal |  | USDA Pullman, WA |
|  | **PI 235668** |  | Netherlands | 668 | USDA Pullman, WA |
|  | PI 326364 |  | Former Soviet Union |  | USDA Pullman, WA |
|  |  |  |  |  |  |
| ***C. glaucus*** | **PI 243151** | BJ-835 | Lebanon | 151 | USDA Pullman, WA |
|  |  |  |  |  |  |
| ***C. palaestinus*** | **PI 235663** | BJ-1964 | Israel | 663 | USDA Pullman, WA |
|  |  |  |  |  |  |
| ***C. leucocaulos*** | **54-53** |  | Knowles collection | 53 | USDA Pullman, WA |
|  |  |  |  |  |  |
| ***C.creticus*** | **UA 060003** |  | Chile | 003 | SemBioSys |
